# Common Errors in Infant Feeding

**Published:** 1910-03-05

**Authors:** 


### Diseases of Children.

### COMMON ERRORS IN INFANT FEEDING.

In a recent issue of the *Clinical Journal* Dr. Eric Pritchard deals exhaustively with the Common Errors in the Feeding of Infants. He strongly advocates the percentage method, and, after 13 years' experience, considers that it is one of the best methods available. It is sometimes stated that the old method is superior to the percentage method, but this, as Dr. Pritchard justly observes, is tantamount to asserting that guess-work is better than calculated certainty, for the percentage enables an accurate degree of dilution being obtained. The percentage method is not suitable for infants under one month of age, chiefly because the young infant's stomach is not yet tolerant to coagulated casein. The author notes an association between hypertrophic pyloric spasm and undue stimulation of the stomach by means of excessive quantities of diluted milk, and is of opinion that cases of pyloric spasm in young children may be prevented from developing into severe forms of stenosis by adequate attention to the feeding. This he secures by providing the newly-born infant with a food which approximately resembles colostrum; Allenbury No. 1 serves this purpose admirably, though Dr. Pritchard prefers simple undiluted whey prepared from fresh milk and rennet. In his opinion no new-born baby should be provided with a wet-nurse until it has served an appropriate apprenticeship on an artificial substitute for colostrum. The fact that a few babies manage to "muddle through," apart from such a probationary experience, is no argument against the adoption of the more scientific, albeit more troublesome, method.

### Test Feeds.

Dr. Pritchard lays stress on the importance of estimating the amount of milk an infant receives at a breast feeding. Apart from the physiological test —namely the progress of the infant, the only means of ascertaining whether an infant receives enough or too little milk is to employ the method to which he has provisionally given the name of the "test-feed." This method consists in weighing the infant before and after feeding on very accurate scales; the amount consumed is estimated by noting the difference in the two weighings. "The physiological test," he writes, "is a very valuable one, and it can be confirmed and substantiated in some degree by noting the amount of urine that is excreted, and by the character and quantity of the stools. Further, the size of the breast before and after feeding affords some sort of index of the amount of milk which has been abstracted by the infant; but in spite of these several indications, I assert with the greatest confidence that there is no man living who can give even an approximately accurate estimation of the amount of milk an infant receives at a breast feeding. Since I have systematically adopted the test-feed I have been forced to modify many of my preconceived views with regard to the nutritional possibilities of the human infant. I find that among breast-fed infants it is not those who are inadequately fed according to accepted scientific data who suffer from malnutrition, but for the most part those who receive an adequate or an excessive amount. I have not had one, but 50 cases, in which the infant has appeared to thrive well and to maintain a good weight curve on a half, and sometimes on a third, of the quantity of food that should be prescribed according to our scientific criteria; for instance, it is quite a common experience to find that an infant three or four months old is fed ten times in the day, and only obtains from 1 to 1½ oz. of milk at a feeding, or about 12 oz. in the 24 hours."

### Food Injuries.

Finally, dealing with food injuries, Dr. Pritchard discusses Finkelstein's theory, and remarks that if proteid matter, owing to excessive quantity, is not digested normally in normal situations, it is digested abnormally in abnormal situations by bacterial agencies, with toxic results that are well known. "Quite apart from considerations of excess of proteid the influence of foreign, or new forms of proteid on the infant organisms, is always a factor that must be reckoned with. Even at the present day, despite the brilliant work of Emil Fischer and his school, there are still whole classes of proteid bodies of the nature of albumoses, polypeptides, and toxins about which we know very little. It is, however, almost certain that many of these bodies are to be found in human milk, and perhaps to a lesser degree in cow's milk, and that some of them possess serious toxic properties. The milk of menstruating women and of epileptics is generally toxic, and before now such milk has proved actually fatal to the infant who has consumed it."

"The ordinary chemical analysis of milk will no more reveal the presence or character of its contained poisons than it will indicate the physiological distinctions between anti-diphtheritic serum and staphylococcic vaccine. I have frequently been called upon to treat breast-fed infants, who have presented severe toxic symptoms although nothing abnormal could be detected by the ordinary methods of investigation in the quantity or quality of the milk; and the same infants have subsequently progressed normally on cow's milk modified to the same chemical standard. Such conditions—and they are extremely common—cannot be due to Finkelstein's fat-injuries, nor yet to his carbohydrate injuries; they are due to albuminoid intoxications, for which at present we have no reliable test, except the somewhat expensive one, the physiological reaction of the infant. To imagine that the chemist with his analytical methods can give a reliable report on the physiological quality of a sample of human milk is to endow him with claims to genius to which he is not at present entitled." Illustrative cases are given of some of the conditions alluded to, and the paper is an able summary on recent work in the feeding of young children.

